# Study on Light Extraction from GaN-based Green Light-Emitting Diodes Using Anodic Aluminum Oxide Pattern and Nanoimprint Lithography

**DOI:** 10.1038/srep21573

**Published:** 2016-02-23

**Authors:** Shengxiang Jiang, Yulong Feng, Zhizhong Chen, Lisheng Zhang, Xianzhe Jiang, Qianqian Jiao, Junze Li, Yifan Chen, Dongsan Li, Lijian Liu, Tongjun Yu, Bo Shen, Guoyi Zhang

**Affiliations:** 1 State Key Laboratory of Artificial Microstructure and Mesoscopic Physics, School of Physics, Peking University, Beijing 100871, China; 2 Beijing North Microelectronics Co., Ltd., Beijing 100176, China; 3 Sino Nitride Semiconductor Co., Ltd., Dongguan 523500, Guangdong, China

## Abstract

An anodic aluminum oxide (AAO) patterned sapphire substrate, with the lattice constant of 520 ± 40 nm, pore dimension of 375 ± 50 nm, and height of 450 ± 25 nm was firstly used as a nanoimprint lithography (NIL) stamp and imprinted onto the surface of the green light-emitting diode (LED). A significant light extraction efficiency (LEE) was improved by 116% in comparison to that of the planar LED. A uniform broad protrusion in the central area and some sharp lobes were also obtained in the angular resolution photoluminescence (ARPL) for the AAO patterned LED. The mechanism of the enhancement was correlated to the fluctuations of the lattice constant and domain orientation of the AAO-pattern, which enabled the extraction of more guided modes from the LED device.

Recently, GaN-based green light-emitting diodes (LEDs) have been attracted much attention because of the application for high efficiency and high quality lighting source. Many researchers concern on the low internal quantum efficiency (IQE) of green LEDs for high content indium in the InGaN active layer. Moreover, a lot of efforts are put on the improvement of light extraction efficiency (LEE) of green LEDs. Much progress has been made in LEE by nanostructures on emission surface of green LEDs^1^,^2^,^3^,^4^,^5^,^6^,^7^,^8^,^9^,^10^,^11^. These structures are fabricated by electron beam lithography (EBL)^1^,^2^, laser interference lithography (LIL)^3^,^4^, nanoimprint lithography (NIL)^5^,^6^,^7^,^8^,^9^, and anodic aluminum oxide (AAO)^10^,^11^. The LEE enhancements for the patterned green LEDs in the above reports range 19% to 25 times compared to the planar ones, depending on the nanostructure parameters and LEE measuring conditions. The EBL technique is the most precise, but also the most expensive and time-consuming method among all, so that it is unsuitable for large scale wafer manufacturing. The LIL method requires costly equipment, which makes it less flexible and time-consuming. Although the NIL is appropriate for a large scale nanostructure manufacturing, the imprinting stamp, usually fabricated by the EBL, is quite expensive, hindering its application from commercial manufacturing. As to the AAO technique, self-organized wet etching of aluminium in spatially regular, triangular order seems to be the simplest and lowest cost one among all the above methods^12^. However, it is disposable and not easy to transfer the AAO pattern to the emission surface directly, especially in mass production^10^,^11^.

A combination of the AAO and NIL techniques may realize the fabrication of nanostructures reproductively and economically. The period of the AAO template can be modified by adjusting the anodization voltages, temperatures, type and concentration of acidic electrolytes^13^. However, the AAO template is not a good stamp for NIL process because of its softness, instability^14^,^15^,^16^ and non-flatness^17^, which cannot stand thousands times of imprinting or imprint large size pattern. Some researchers evaporate the Al films on the Si substrate or GaN films, then anodize Al films as the mask to etch the substrate^17^,^18^,^19^. The AAO patterns suffer from the complicated surface states (roughness and crystallite sizes) and nonuniformity of the deposited Al film^17^,^18^. Moreover, Si substrate is too fragile to stand numbers of imprinting process. So it is important to find a reproductive method to make use of the AAO template as imprinting stamp. Furthermore, some reports show that a larger lattice constant of photonic crystal (PhC) can increase the possibility of extracting guided modes from LEDs^20^,^21^,^22^. However, it is difficult to fabricate large area AAO template with lattice constant over 500 nm due to unstable anodization at relatively high applied voltage above 200 V^13^. Aluminium oxalate hydrate is employed to overcome the burn-through issue in the high-voltage anodization^23^. More anodization conditions and thermal dissipation should be well controlled to obtain large lattice constant.

In addition, thickness of epilayer and lattice constant, pore diameter, and depth of the two-dimensional (2D) PhC have been studied on the light extraction from LEDs^20^,^21^,^22^,^24^,^25^,^26^. The maximum of LEE without encapsulation is achieved as 73% in an ultrathin LED^21^. And the guided modes are coupled with the reciprocal lattice vector of PhC, which is revealed by angle-resolved luminescence spectra and analysed by Ewald construction and calculation^20^,^26^. It is notable that a few guided modes and exact PhC pattern are coupled. For a conventional LED, dozens of guided modes are difficult to be extracted out completely. AAO patterns are gradually used to extract more modes from LEDs. However, there are no clear pictures about the guided mode extracted from AAO pattered LED surface. Some researchers only consider the light emitting from the top of the LEDs when measuring the enhancement of the AAO structure, which cannot understand the extraction process without examination the coupling modes extracted out from the AAO pattern^10^,^11^,^19^. Therefore, it is necessary to develop an accurate measurement to investigate the light extraction mechanism of quasi-period PhCs structures using AAO pattern.

In this paper, the quasi-period PhCs pattern of AAO template was transferred onto a two-inch sapphire substrate by NIL and induced coupled plasma (ICP) etching. The AAO patterned sapphire substrate can serve as the nanoimprinting stamp, which is much more economic and reproducible. The quasi-period PhCs patterns were imprinted on the surface of the GaN-based green LEDs by AAO sapphire stamp. The green LED was grown by metalorganic chemical vapor deposition (MOCVD) on the double polished c-plane patterned sapphire substrate (PSS), for which the period was 3 μm, the diameter was 2.2 μm, and the height was 1.5 μm. The LED structure was consisted of 2 μm undoped GaN, 2 μm n-type GaN, five quantum wells (QWs) (InGaN (2.5 nm)/GaN (12.5 nm)) and 150 nm thick p-GaN layer. The peak wavelength was 545 nm. The schematic structure of LED is shown in Fig. 1. There were three samples with different pore depths on the LED surface: 100, 130 and 150 nm (called AAO_100 nm, AAO_130 nm, and AAO_150 nm). As reference, three exact period PhCs samples were similarly fabricated as PhC_100 nm, PhC_130 nm, and PhC_150 nm respectively by a specific stamp which made by EBL. The optical properties of quasi and exact period PhCs on the green LEDs were investigated by photoluminescence (PL), PL microscopy, angular resolution PL (ARPL) measurements and finite difference time domain (FDTD) simulation. Combined with the Ewald constructions, the guided modes extracted from green LEDs by AAO pattern were demonstrated. Significant enhancement of the light extraction from PSS LED can be achieved by AAO structures, which adds more commercial value.

## Results

A two-inch AAO template with lattice constant of 520 nm was obtained by high anodized voltage above 200 V, low concentration phosphoric acid solution, and good thermal dissipation. Figure 2 shows the images of two-inch large area AAO template and its replicas on a sapphire substrate and a polymer membrane. Figure 2a shows a colourful diffraction phenomenon on the AAO template. The continuously varied colour corresponds to the different diffraction direction of the white light. Figure 2b shows the top-view scanning electron microscopy (SEM) image of the prepared AAO template. The inset shows the histogram of AAO pattern’s dimension distribution. The pattern shows well-aligned and triangularly packed nanopore arrays with pore dimension of 375 ± 50 nm. Some imperfections are also shown at the boundary of the nanopore array domains. The pattern of the AAO template was transferred to the sapphire substrate by nanoimprint lithography and ICP etching with Ni mask. AAO patterned sapphire stamp was imprinted into a polymer membrane to form an intermediate polymer stamp (IPS). Figure 2c shows the image of two inch IPS with AAO pattern. The IPS also shows a colourful diffraction phenomenon just as the AAO template. Although the AAO pattern was perfectly transferred onto the sapphire substrate, which is shown in Fig. 2d, the colourful diffraction of sapphire stamp was very weak because of its transparent bulk characteristic (not shown here). Figure 2e shows the cross-sectional SEM image of the AAO patterned sapphire stamp. The depth of nanopores is about 450 ± 25 nm. The inclined sidewall is sharp. Both the surface and the bottom are very smooth. The atomic force microscope (AFM) image of the sapphire stamp is also shown in Fig. 2f. The inset is the cross-sectional profile along the red dash line in Fig. 2f. A nonflatness of below 5 nm can be achieved in the area of 5 μm × 5 μm on the top surface. The less fluctuation of pore depth and the extremely smooth surface is suitable for the stamp usage.

Figure 3 shows the top-view SEM images of AAO_130 nm and PhC_130 nm samples. Similar to AAO sample, the lattice constant and diameter of the nanopores were 520 and 375 nm for the PhC_130 nm LED sample. The insets show the fast Fourier transform (FFT) of the SEM images, which are consistent with the diffraction patterns of the structure^27^. The ring-shaped pattern of the AAO-LED indicates that the nanopore arrays show an amorphous structure, while the six-fold symmetry spots array pattern in the PhC-LED reveals a triangular periodic structure. The radius of the ring for AAO-LED is similar to the length of reciprocal lattice vector for PhC-LED. It indicates that the AAO pattern is something short range triangular order. In long range, the anodized domains distribute in orientation randomly. The width of the ring is related to the period distribution which is mainly due to the fluctuation of the anodization voltage. The lattice constant of the nanopores in the AAO pattern can be determined as 520 ± 40 nm.

So, in our work, the variation of AAO pattern can be constrained in a certain range due to stability of the anodization process. Besides, the AAO pattern was transferred by nanoimprint lithography, so that the pattern on the surface is exactly the same as the original nanoimprint stamp. Furthermore, the as-fabricated sapphire nanoimprint stamp can stand thousands times of imprint process, which doesn’t need to fabricate AAO template frequently. At last, materials used in our work, such as aluminum and sapphire, are low-cost.

The PL spectra of the AAO- and PhC- LEDs with different pore depths were measured in an integrating sphere system, as shown in Fig. 4. The light output power (LOP) enhancements of the AAO-LED samples are 82.2%, 116.3% and 102.2% for AAO_100 nm, AAO_130 nm and AAO_150 nm samples respectively, in comparison to planar LED. The enhancements of the PhC-LED samples are 67.9%, 106.3%, and 82.2% for PhC_100 nm, PhC_130 nm, and PhC_150 nm samples, respectively. The maximum enhancements for AAO-LED and PhC-LED are higher than the results of 45%^23^ and 94%^19^ for AAO-LED reported in other work. With the etching depth increasing, more guided modes are coupled with the nanostructures and become leaky modes^24^,^26^, which enhances the light extraction from LED. However, lattice damage, caused by dry etching process, would introduce deep levels in the active region that serve as non-radiative recombination centers, leading to the deterioration in optical properties of a GaN-based LED^24^. If the etching damages are recovered by KOH treatment and the holes are filled with ITO layers, the electrical properties would not degrade^23^,^28^. In addition, there is a blue shift for the PL peak wavelength for all the LEDs after surface patterning because the compressive stain is relaxed during the etching process. According to the LOP enhancements of two types LEDs, the quasi-period AAO pattern extracts more light from LED than that from the exact period one.

Besides the integrating PL measurement, the emission patterns were also measured to study the light extraction from the green LEDs with AAO- and PhC- structures. When the laser beam was coupled into the light path of the microscope, the laser spot was about several microns on the samples. The photograph taken in the microscope stands for the far-field pattern projected on the image plane approximately. Figure 5a–c shows the emission patterns of planar, PhC_130nm and AAO_130nm LEDs. The PSS arrays of all the samples are lightened up because the condition of total internal reflection is broken. The bright spot arrays are not diffraction spots according to the periodicity of 3 μm for PSS. The emission contrasts of the AAO_130 nm and PhC_130 nm samples are much brighter than that of the planar one. More PSS areas are lightened up for the patterned samples too. It is reasonable that both the AAO and PhC structures achieve obvious improvements of light extraction. A hexagonal symmetry is observed from the emission pattern of the PhC_130 nm sample, which is marked by white arrows in Fig. 5b. As to the AAO_130 nm sample, the emission pattern is a deteriorate hexagon due to the quasi-period PhC structures (also marked in Fig. 5c). Higher brightness is observed on the left side of the AAO emission pattern. The asymmetry of the emission pattern can be due to the fluctuation of domain orientation of AAO structures. There are more bright areas in the approximate far field pattern for AAO samples than that for PhC ones, which indicates that more guided modes can be extracted by AAO structures. The emission pattern in the Fig. 5a seems to be a round one, which cannot be marked in hexagon.

The PL emission pattern taken in the microscope mainly corresponds to the distribution in the azimuth direction. To get better insight into the light extraction in the zenith direction, the ARPL spectra of planar, AAO- and PhC- LEDs have been measured, as shown in Fig. 5d,e. It is notable that the symmetrical patterns are observed in Fig. 5d, which is different with PL microscope image shown in Fig. 5c. The difference can be explained by the fluctuation in different measurement dimensions. The laser spot in ARPL measurement is millimeter-scale, while that for PL microscope is about several microns. The angular plots of all the samples show broad shape protrusions in −30 ~ 30^o^. The PL intensities of the AAO_130nm and PhC_130 nm samples are about 2.38 times and 2.35 times higher than that of the planar one in the perpendicular direction, as shown in Fig. 5d. Furthermore, it is found that the angular plots of AAO-LEDs show much smoother than those of PhC-LEDs in the central protrusions. The less diffraction fringes are also observed for AAO_130 nm sample in Fig 5e. There are some lobes when the incidence angle θ is around 60 ~ 70° for AAO-LEDs. The enhancement of PL intensity can be induced by Bragg scattering, which is directly related to the extraction efficiency^29^. Therefore, more modes light could be extracted out from the quasi-period PhC pattern on the surface of AAO-LED, which consists of a wider range of lattice constants and more orientations than those from the exact period pattern of PhC-LED. These extracted modes overlap each other, generating the smooth plot in the central region. The large lobes at 60 ~ 70° can account for the fluctuation of domain orientation and lattice constant of AAO structures. The details will be discussed later.

## Discussion

**Figure 1:**
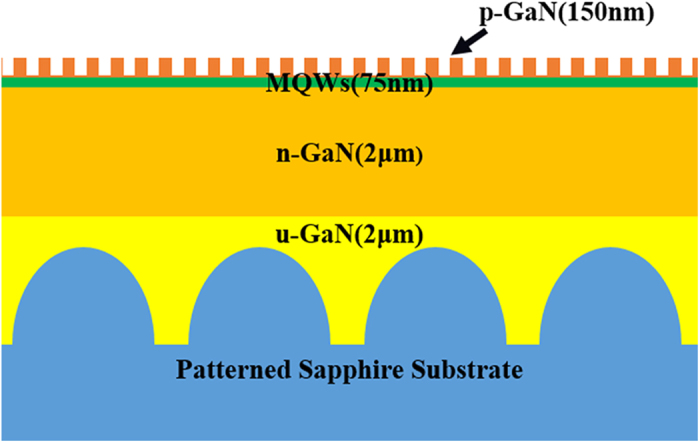
The schematic structure of LED. The green LED was grown on the double polished c-plane PSS, for which the period was 3 μm, the diameter was 2.2 μm, and the height was 1.5 μm. And it was consisted of 2 μm undoped GaN, 2 μm n-type GaN, five QWs (InGaN (2.5 nm)/GaN (12.5 nm)) and 150 nm thick p-GaN layer.

**Figure 2:**
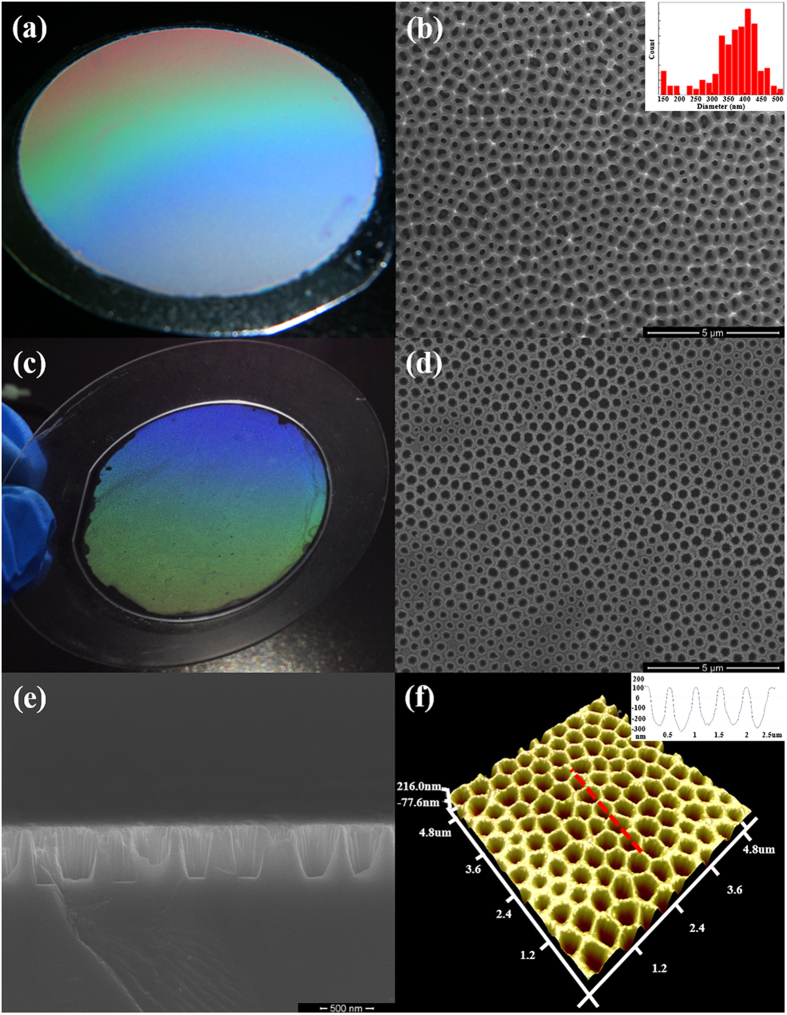
(**a**) Two-inch large-area AAO template; (**b**) top view SEM image of the AAO template; The inset shows the histogram of AAO pattern’s dimension distribution; (**c**) the IPS imprinted by two-inch sapphire nanoimprinting stamp; (**d**) top view and (**e**) cross-sectional SEM images of the sapphire nanoimprinting stamp; (**f**) AFM image of the sapphire nanoimprinting stamp and the inset is the cross-sectional profile along the red dash line. Both the AAO template and the IPS show colourful diffraction phenomena. The pore dimension of the AAO template is 375 ± 50 nm. The pattern on the surface of sapphire stamp highly coincides with the one on the surface of AAO template. The nanopores have trapezoid sidewall and flat bottom. The etching depth is about 450 ± 25 nm. Therefore, the sapphire surface nonflatness of sub-5 nm can be observed.

In order to investigate the mechanism of the light emission enhancement, the LEE of LEDs with different patterns and pore depths were calculated by three-dimensional (3D) FDTD. The simulation surface structure of AAO-LED is simplified from the above SEM images, as shown in Fig. 6a. And the cross-sectional structure is shown in Fig. 1. Figure 6b reveals the enhancement of the LEE at the various pore depths for AAO-LEDs and PhC-LEDs. The highest enhancement of LEE, 87.4%, for the AAO-LED is obtained when the pore depth is 130 nm. It is 80.6% for the PhC-LED at the same depth, consistent with the PL results. The distinction between simulation and experiment results should be due to the offset between the simulation structure and the actual structure for the side-wall angle and small simulation area limited by computation. When the simulation area is 6 μm × 6 μm, the ratio of the height to the width of the simulation model is so big that more light would propagate through the sidewall, resulting in a lower LEE. More complicated model and larger simulation area would yield more accurate results. Anyway, the LEE enhancements by AAO and PhC patterns are illustrated accurately. Furthermore, 130 nm pore depth is optimized for a 150 nm thick p-GaN layer and the dimension of PSS used in this work. The pore depth should be optimized according to the thickness of p-GaN layer and the shape of PSS in other samples.

Top-view far-field patterns of PhC-LED and AAO-LED are shown in Fig. 6c,d, respectively. The simulation area is 6 μm × 6 μm, which is similar with the size of the laser spot in the PL microscope measurement. A six-fold symmetry is observed in the far-field distribution of the PhC-LED, consistent with the hexagonal pattern in the PL microscope measurement. Besides, the peak positions of light extraction are distributed in several discrete zenith angles, which are mainly within 40°. That is the reason that some broad-shaped fringes are measured in the central area by ARPL. Regarding AAO-LED, the peak positions of light are distributed not only within a narrow angle in the zenith direction, but also in higher angles around 60 ~ 70°, which is consistent with the result of ARPL. Moreover, six-fold symmetry is also destroyed and more brightness is observed on the left side of the AAO far-field pattern, which is consistent with the image taken by PL microscope. So the simulated far-field patterns can describe the emission pattern observed in PL microscope precisely.

The number of guided modes in a GaN-based LED increases with the thickness of the GaN film^20^,^21^. So there should be dozens of guided modes existing in this work. The extraction of these guided modes light into the air is determined by the Bragg diffraction condition


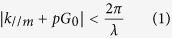


where, k_//m_ is an in-plane wavevector corresponding to *m*^*th*^ guided mode, *p* is an integer, *G*_*0*_ is the reciprocal lattice vector defined as 2π/*a*, *a* is the lattice constant of PhCs, and *λ* is the wavelength of light. The Bloch mode is then transferred to a leaky mode when the Bragg diffraction condition is satisfied.

**Figure 3:**
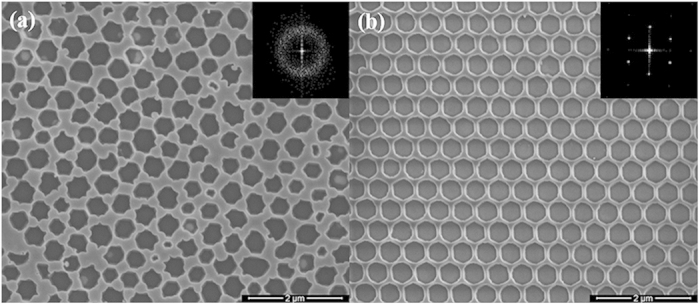
Top-view SEM images of (**a**) AAO_130 nm sample and (**b**) PhC_130 nm sample. The insets show the FFT of the SEM images. The pore arrays of AAO-LED show an amorphous structure, while those for PhC-LED show the 2D crystalline one. The lattice constant of the AAO template is 520 ± 40 nm.

**Figure 4:**
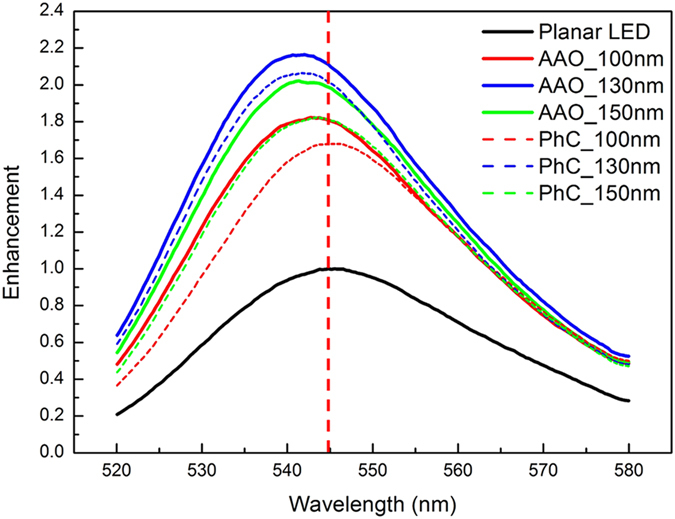
PL spectra measured by the integrating sphere system. Obvious improvements in light output for all the AAO-LEDs and PhC-LEDs are achieved. The maximum enhancements are obtained at the pore depth of 130 nm. Besides, a blue shift of peak wavelength is observed.

**Figure 5:**
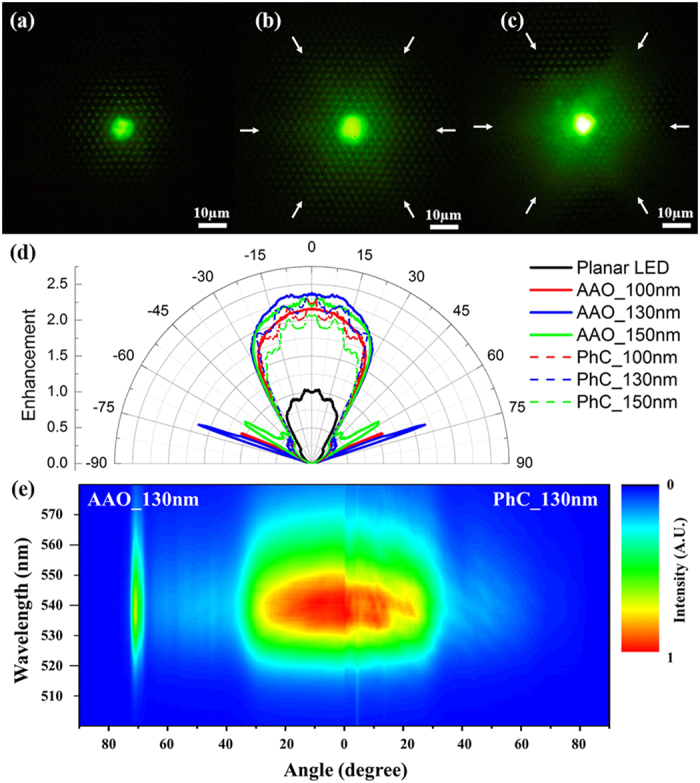
Top-view photographs of (**a**) planar LED, (**b**) PhC_130nm sample, and (**c**) AAO_130nm sample taken in PL microscope. (**d**) Emission patterns for planar, AAO- and PhC- LEDs; (**e**) angular resolution PL spectra for AAO-LED (left) and PhC-LED (right). Both of them were measured from positive zenith. The luminescence from the AAO_130 nm and PhC_130 nm sample is much brighter than that from the planar LED. Besides, the pattern of luminescence from the PhC_130 nm sample is a prefect hexagon while the one from the AAO_130 nm sample is a deteriorate hexagon, which is marked by white arrows. All of the AAO-LEDs and PhC-LEDs gain the light extraction enhancements compared with the planar one. Moreover, the angular plots of AAO-LEDs show much smoother than those of PhC-LEDs in the central protrusions and a few sharp lobes are observed when the zenith angle θ is around 60 ~ 70°.

**Figure 6:**
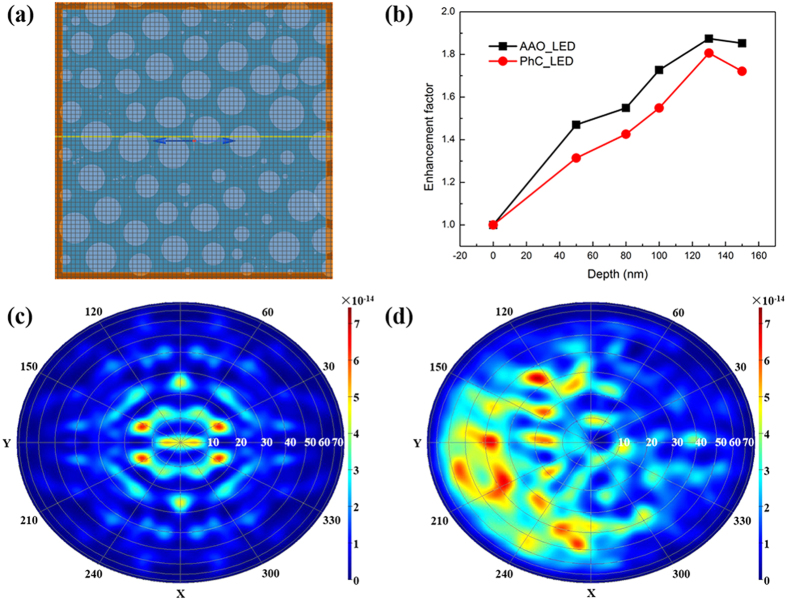
(**a**) Schematic structure of AAO-LED for FDTD simulation; (**b**) calculated light extraction enhancements of AAO- and PhC-LEDs with different pore depths using 3D FDTD. Far-field emission pattern projections from (**c**) PhC-LED and (**d**) AAO-LED. A six-fold symmetry is observed in the far-field pattern of the PhC-LED, but not in the AAO-LED. Therefore, the light in the PhC-LED mainly distributes inside narrow zenith angle while more light is extracted from high zenith angle in the AAO-LED. The far-field pattern of the AAO-LED is affected by the fluctuation of the domain orientation in the simulation area.

**Figure 7:**
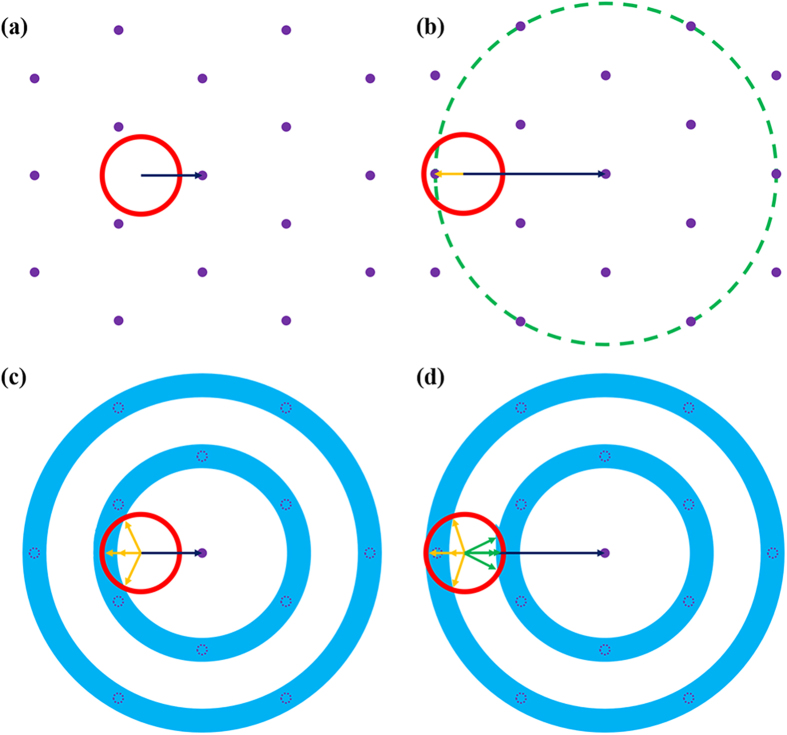
(**a,b**) Ewald constructions of a triangular PhC for different modes, while (**c**,**d**) Ewald constructions of a quasi-period PhC for different modes. The red circle describes the extraction cone. The blue arrow describes a guided mode. The yellow arrows and green arrows describe the harmonic falling in the extraction cone. It is more potential for a guided mode to satisfy the Bragg diffraction condition in a quasi-period PhC structure than that in an exact PhC structure.

According to refs 21 and 26, the Ewald constructions are shown in Fig. 7. The purple dots represent the reciprocal lattice points of the triangular PhC in Fig. 7a,b, which is consistent with the FFT image in the inset of Fig. 3. The red solid circle is the extraction cone. If the resultant wavevector falls into the circle, the guided mode will be extracted to the air. For the guided mode (blue arrow) in Fig. 7a, there are no reciprocal lattice vectors to be satisfied for Eq. 1. The guided mode in Fig. 7b can find a reciprocal lattice vector to fall into the extraction cone. Besides, reciprocal lattice points would become a circular locus (dashed circle in Fig. 7b) when the azimuthal angle of the guided mode is changed. The arc of the circle inside the extraction cone can be diffracted. Therefore, some guided modes would satisfy the diffraction condition and enhance the coupling efficiency under different propagation directions. The extraction of other guided modes would be suppressed ascribing to being dissatisfied with the Bragg condition at the same time. All these guided modes exhibit their own positions in spatial distribution. So the emission pattern in the PhC-LED can be optimized by altering the lattice constant, filling factor and etching depth^24^. The far-field emission pattern of PhC-LED is modified by the PhC diffraction, which shows several fringes of the main protrusion in the zenith angle range of −30 ~ 30^o^, as shown in Fig. 5a. It indicates that some special guided modes, whose wavevectors satisfy the Bragg condition, are diffracted by PhC structure with only one specific lattice constant, due to the discrete nature of guided modes.

The Ewald constructions for AAO structures are also shown in Fig. 7c,d. According to the diffraction pattern deduced from FFT of SEM images of AAO-LED in Fig. 3, triangular reciprocal lattices are substituted by a serial of concentric rings. The purple dot circles in the rings represent the reciprocal lattice point positions in Fig. 7a,b. It is obvious that the guided modes, which cannot be extracted by PhC, can be extracted by AAO structures, as shown in Fig. 7c. A crescent moon shape (yellow arrows marked) is formed in the overlap of the extraction cone and the nearest ring, which indicates the increasing extraction probability of the guided modes into the air. A large k_//_ mode, which can be extracted by PhC, can also be extracted easily by AAO structures. In Fig. 7d, the extraction cone overlaps with not only the second nearest ring in a large crescent moon shape region (the yellow arrows marked), but also the nearest rings in a certain part (the green arrows marked). Therefore, the Bragg diffraction condition is satisfied for many guided modes, maybe some successive ones, leading to a more uniform main protrusion in the far-field emission pattern. Besides, the sharp lobes at the zenith angles 60 ~ 70° in the emission pattern also indicate that some guided modes are diffracted from the AAO-LED rather than the PhC-LED. The extraction of these guided modes is influenced by the etching depth of the AAO structures. These leaky modes can clearly explain the enhancement in the light extraction of AAO-LEDs. However, the coupling efficiency between the guided modes and AAO pattern is lower than that of PhC in this work. The coupling efficiency would be enhanced by controlling the anodization conditions and adjusting the distribution of quasi-period PhC parameters and domain orientations.

Nowadays, surface roughening, achieve through simple process, has been widely used in commercial GaN-based LED manufacturing^30^. However, its pattern is uncontrollable and the light in the LED can be extracted from the micro-scale pattern by geometrical optics. Although both of AAO and PhC templating methods are nano-scale pattern and extract more light out by coupling with the reciprocal lattice vector, AAO templating method would achieve a quasi-period PhC structure and extracted more guided mode from the LED. So, AAO templating method is promising for commercial manufacturing due to its excellent properties and low-cost.

## Conclusion

In summary, the AAO template, with the lattice constant over 500 nm was fabricated by a two-step anodization. It was successfully replicated onto the surface of sapphire substrate. The AAO patterned GaN-based green LEDs with different pore depths have been fabricated using AAO sapphire stamp. The improvements in LEE of the AAO-LEDs were similar to those of the PhC-LEDs, while the AAO-LEDs showed uniform broad protrusion in the central areas and some sharp lobes in far field emission pattern. The FDTD simulation suggested that more guided modes could be extracted by the quasi-period PhC structures with fluctuant lattice constant and domain orientation. The Ewald constructions further demonstrated the extraction mechanism of the guided modes by AAO structures.

## Methods

### Samples preparation

The electro-polishing treatment was implemented onto the surface of Al foil (the purity over 99.999%) in a mixture of HClO_4_ and C_2_H_5_OH (HClO_4_:C_2_H_5_OH = 1:4, volume ratio). Anodization current would extend homogeneous in a robust Al foil due to its good crystal quality. And the surface of Al foil was flat after the electro-polishing treatment, which is necessary for pattern transferring. The backside of Al foil was well sealed up and in direct contact with the coolant liquid in our apparatus. The AAO sample was formed by a conventional two-step anodization, which was used to obtain self-organized arrays with good regularity and circularity^12^. Masuda *et al.* reported that the lattice constant had a good linear relationship with the applied voltage, where the proportionality constant of cell size per applied voltage was approximately 2.5 nm/V^13^. The anodization was performed in 0.01mol/L phosphoric acid solution at 210 V during 0 °C for 10 h to obtain highly ordered nanopore arrays. Then the irregular AAO film was removed in a mixed solution with 2 wt. % chromic acid and 5 wt. % phosphoric acid. The second anodization process was performed for 15 min in the same conditions with the first anodization, and then widened the pore in a mixed solution with 2 wt. % chromic acid and 5 wt. % phosphoric acid for 5 min.

After that, an antistick treatment was applied to the AAO template by spin-coating fluorinated silane. In the meantime, a 150 nm-thick Ni layer was deposited on a c-plane sapphire wafer by electron beam evaporation. The use of tackifier as an adhesion layer prevented the resist peeling-off from the Ni layer during the imprinting process, followed by spin-coating of the resist onto the surface. The AAO template was employed as the imprinting stamp and a two-step simultaneous thermal and ultraviolet curing (STU) imprinting process was applied using an Obducat Eitre® 3 Nanoimprinting instrument. The resist spin-coated onto the Ni layer must be thick enough and the time of nanoimprint lithograph process should be long enough to fill the space among the IPS. Otherwise, the resist would just accumulate at the edge of pillar patterns on the IPS and nano-ring or some other unexpected patterns would be obtained^31^.

**Figure 8:**
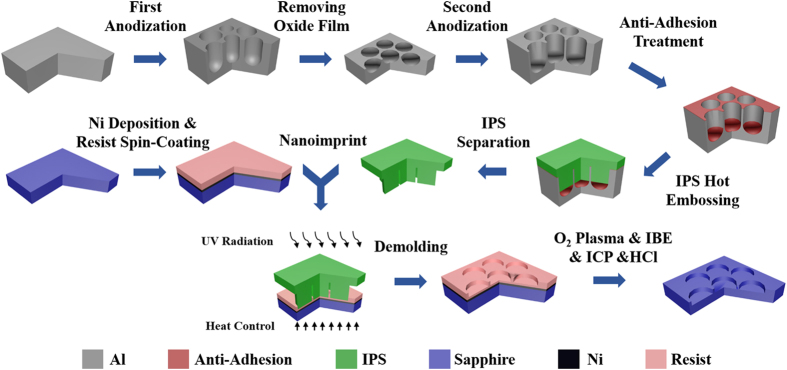
The flow diagram of sapphire nanoimprinting stamp manufacturing. Ni is chosen as a hard mask during the ICP process to gain an applicable depth on the surface of sapphire.

When the residual resist was removed by O_2_ plasma, the Ni was etched by ion beam etching (IBE, IBE-150B), resulting in the pattern of AAO template transferred to Ni layer. Then the sapphire was etched with Ni hard mask using BCl_3_ (100 sccm with the chamber pressure kept at 2.2 mTorr) by ICP (ELEDE380). The ICP power and bias power were set at 1400W/400W. Finally, the Ni layer was removed by hydrochloric acid and an antistick treatment was applied to it by spin-coating fluorinated silane. The processes of AAO patterned sapphire stamp are shown in Fig. 8.

The green LED wafer used in this work was grown by MOCVD on the double polished c-plane PSS with five QWs (InGaN (2.5 nm)/GaN (12.5 nm)) and 150 nm thick p-GaN layer. The peak wavelength of the emitted light was 545 nm. A 100 nm-thick SiO_2_ layer was deposited on the sapphire wafer at 250 °C by plasma-enhanced chemical vapor deposition (PECVD), followed by spin-coating of the resist onto the surface. The AAO sapphire stamp was employed and a two-step STU imprinting process was applied using an Obducat Eitre® 3 Nanoimprinting instrument. When the residual resist was removed by O_2_ plasma and the SiO_2_ was etched by reactive ion etching (RIE) with CHF_3_ and O_2_, the pattern of AAO template was transferred to SiO_2_ layer. Then the p-GaN layer was etched with SiO_2_ mask using a gas mixture of Cl_2_/BCl_3_/Ar by ICP for three different pore depths: 100, 130 and 150 nm. At the same time, the samples with exact period PhC structure and the same depths were prepared using triangular order nanoimprinting stamp in the same process. Finally, potassium hydroxide solution was used to remove the etching damage for all samples and the SiO_2_ layer was removed by BOE (NH_4_F: HF = 7: 1).

### Characterization methods

The surface of the AAO template, sapphire stamp and GaN-based LED samples were carefully studied by SEM (Nova Nano SEM 430) and AFM (Bruker Dimension Icon with ScanAsyst). The enhancements of light extraction from GaN-based green LEDs were investigated by PL using an integrating sphere system. The effects of the PhC structures on LED performance were characterized by PL microscope and ARPL. The samples were excited by a 405 nm laser (DL-405-200–T), and related spectra were recorded using a spectrometer system (Ocean Optics, USB2000 + ). For ARPL measurements, the angular resolution was 0.5°.

### FDTD simulations

The simulations were calculated by 3D FDTD solutions tools (Lumerical FDTD Solutions, Inc.). All of the structure parameters were taken from the data mentioned above. The surface structures came from the measured SEM images, as shown in Fig. 3. The SEM image was converted to binary after the binarization process so that we can distinguish the pores from the surface. And the original pattern on the surface of AAO-LED was substituted by the round pattern in the same area. The center of the round is the same as the centroid of the original pattern. The depth of pore is the same and the sidewall is vertical. According to our previous work, the integrated transmission of conical pillar arrays was only 11.3% higher than that of the cylindrical pillar arrays^32^. So the etching sidewall was set as vertical in our FDTD simulation since the effect of the sidewall angle is not significant. A dipole with a wavelength of 545 nm was put at the location corresponding to the MQWs. The GaN was set to be transparent. The simulation area was 6 μm × 6 μm.

## Additional Information

**How to cite this article**: Jiang, S. *et al.* Study on Light Extraction from GaN-based Green Light-Emitting Diodes Using Anodic Aluminum Oxide Pattern and Nanoimprint Lithography. *Sci. Rep.* **6**, 21573; doi: 10.1038/srep21573 (2016).
